# A novel scale for anxiety-related fixation instability during laser in situ keratomileusis

**DOI:** 10.1038/s41598-025-92048-y

**Published:** 2025-03-18

**Authors:** Hazem Abdelmotaal, Magdi Mohammad Mostafa, Ahmad Abd El-Nasser Awad, Zeiad Eldaly, Mahmoud Abdel-Radi

**Affiliations:** Department of Ophthalmology, Faculty of Medicine, Assiut University Hospital, Assiut University, 6th Floor, Assiut, 71516 Egypt

**Keywords:** Laser in situ keratomileusis, Anxiety, Hospital Anxiety and Depression Scale, Fixation instability, Eye-tracker, Medical research, Eye diseases

## Abstract

This cohort study aimed to investigate the correlation between the severity of anxiety during laser in situ keratomileusis (LASIK) and fixation instability, as measured and plotted by the eye tracker during photo-ablation, and to develop a novel quantitative scale for anxiety-related fixation instability. LASIK was performed to correct myopia and hypermetropia with and without astigmatism in 2435 eyes of 2435 patients. Participants fulfilled the seven-item anxiety sub-score of the Hospital Anxiety and Depression scale questionnaire for scaling patients’ anxiety levels before LASIK into normal, borderline, and anxiety case groups. The eye-tracking pupil center position plots, patient’s heart rate, and surgeon-reported level of patient’s cooperation during the procedure were analyzed. An anxiety-related fixation instability score (FIS) was calculated, for which the best cut-off points to differentiate between normal, borderline, and anxiety case groups were defined. The FIS showed a high performance in separating participants into normal, borderline, and anxiety case groups and when used as a scale (0–90), values from 0 to 12 are considered normal, from 13 to 36 are considered borderline, and from 37 to 90 are considered anxiety cases. The FIS and scale are useful objective tools to quantify anxiety-related fixation instability during LASIK.

## Introduction

Anxiety is common among patients undergoing eye surgery, which is typically performed under topical anesthesia while the patient is awake. Patients may also experience anxiety due to concerns about surgical pain, possible complications, and the uncertainty of the outcome of the surgery^1,2^.

Fixation instability is a common problem during LASIK surgery and may lead to unexpected outcomes^3^. Eye movements can have a large amplitude, a frequency of over 100 Hz, and a corneal speed of around 150 mm/s^4^. With the advancement of scanning-spot excimer lasers, much work has focused on increasing the efficacy of the photo-ablation and smoothness of the ablated surface, especially with respect to the position of the patient’s eye by developing state-of-the-art eye trackers to help maintain alignment during photoablation to improve refractive outcome^5^. Excimer laser devices are equipped with ultrafast, high-latency, and multidimensional tracking functionality that uses the pupillary center (PC) as a landmark to maintain intraoperative alignment of the photoablation. An ablation treatment report can include a pupil position plot summarizing the PC position offsets relative to the fixation target based on the regularly sampled PC shifts during ablation^6^.

In this study, we aimed to investigate the correlation between the severity of anxiety symptoms during LASIK, and fixation instability during photo-ablation as plotted by the eye tracker. Furthermore, we developed a novel quantitative scale for anxiety-related fixation instability.

## Methods

### Study design and participants

This was a prospective, non-consecutive, observational, cohort study conducted at two eye-specialty centers from August 2018 to July 2023. All patients were informed about the risks and benefits of the procedure and provided written informed consent. The study included candidates undergoing microkeratome-assisted laser in situ keratomileusis (MK-LASIK) and femtosecond laser-assisted laser in situ keratomileusis (FS-LASIK) for correction of myopia and hypermetropia with and without astigmatism. Patients, willing to participate in the study, were included if they were aged ≥ 18 years. Maximum corrections were 12.0 diopters (D) for myopia, 6 D for hyperopia and 6 D for astigmatism. For all eyes, corneal thickness at thinnest location was ≥ 500 µm with an estimated postoperative residual stromal bed of at least 60% of the thinnest pachymetry^7^. Exclusion criteria were previous corneal surgery, pregnant or lactating females, and concomitant ocular or systemic diseases that contraindicated LASIK. Also, patients with communication barriers, hypochondria, previous stressful surgical experiences, not necessarily related to ophthalmic procedures or taking psychotropic or anxiolytic drugs, or having a history of any clinically relevant psychiatric or cardiovascular disease were excluded.

Preoperative assessment included a detailed history taking and a complete ocular examination including Scheimpflug-based corneal tomography.

### Subjective assessment of anxiety

The standardized clinician-related Hospital Anxiety and Depression scale anxiety sub-score (HADS anxiety sub-score) was utilized for the assessment of anxiety to ensure completeness of reporting. The HADS questionnaire was originally developed by Zigmond and Snaith^8^. HADS is a fourteen-item scale with seven items for each of the anxiety and depression sub-scores. We focused on the use of the seven-item anxiety sub-score for scaling a patient’s anxiety levels, in an Arabic-translated form of the questionnaire provided by the HADS copyright holders, before LASIK. Patients were asked to read a statement (e.g., “worrying thoughts are going through my mind”) and underline the most appropriate of the following responses: a great deal of time, a lot of time, from time to time but not often, or only occasionally. The responses were scored as 3, 2, 1, and 0, respectively and the overall score for all 7 HADS-Anxiety questionnaire items was summed to get the HADS anxiety sub-score. A sub-score ≤ 7 denotes no anxiety scale (normal), a sub-score of 8–10 denotes doubtful anxiety scale (borderline), and a score of 11–21 denotes a definite anxiety scale (case).

None of the patients received any systemic sedatives or anxiolytics in the pre or intraoperative periods avoiding potential confounding effects on measured variables.

### Objective assessment of anxiety

Heart rate (HR) was used as an objective measurement of anxiety. Baseline HR was measured following ophthalmic examination, while patients were comfortably positioned supine to minimize potential effects of postural changes on heart rate measurements during the subsequent surgical procedure.

Upon entering the operating room, patients were attached to a pulse oximeter while lying in a supine position, and an operating room nurse recorded the HR at the start of the operation, during flap creation, during ablation, and at the end of surgery. Blood pressure was not included as an additional parameter to avoid starting the patient with sudden inflation of the sphygmomanometer cuff, which could contribute to stress and cause inadvertent movements.

### LASIK procedure

Patients received a detailed explanation of what they might experience during surgery, and the need for their cooperation expecting periods of darkness, moving instruments and surgeon hands, machine noise, abnormal smells, and irrigation fluids. For participants, undergoing bilateral surgery only the first eye receiving laser correction was included in the analysis to avoid the effect of the patient’s first eye experience. We used a random number generator to determine which eye to start with if there was no specific preference from the surgeon. The flap creation technique was chosen according to the surgeon’s and patient’s preferences after a thorough explanation of the risks and benefits of each technique. Local anesthesia was achieved by preservative-free oxybuprocaine hydrochloride 0.4% drops. Moria 2 Microkeratome (Moria SA, Antony, France) and Wave Light FS-200 femtosecond Laser (Alcon labs, Fort Worth, TX, USA) were used to create the flap in MK-LASIK and FS-LASIK, respectively. Laser ablation was performed using WaveLight EX500 Excimer Laser (Alcon labs, Fort Worth, TX, USA) with patients fixating towards a green intermittent central light throughout the whole procedure. Two experienced surgeons (H.A and M.A) performed LASIK correction of all enrolled eyes.

### The eye-tracking pupil position plot

The WaveLight EX500 Excimer Laser features a multi-spatial 1050 Hz-type eye tracker (synchronized at 500Hz) with latency times as short as 2.5 ms that includes an active, video-based, closed-loop tracking scheme. It’s this scheme that allows the system to capture the image, process it, and then verify eye position with the next image before releasing the pulse. After treatment, the system releases a summarized treatment report including a plot that depicts various pupil center positions within 3.0 mm off-center (on x and y axes) relative to the eye tracking cameras during ablation. The complete pupil center movement per surgery time data can be supplied by a special manufacturer’s software and wasn’t available in our study (personal communication with Alcon).

### Evaluation of patient’s cooperation by surgeon

The patient’s cooperation during the surgery period was graded by the surgeon on a 10-point Visual Analogue Scale (VAS), with 1, totally cooperative (calm and steady fixing) and 10, totally uncooperative (marked eye and head movement and/or lid squeezing).

### Data collection and processing

The following fully automated processing steps were performed using custom Python scripts and the OpenCV library (version 4.5.4, http://opencv.org).

1. Treatment reports were exported from the WaveLight EX500 as portable document format (PDF) files for all studied patients. The custom Python script selected only the studied eye of every participant with its data relevant to our study and organized them in a data frame that includes patient ID, gender, birth date, treatment date, refractive and corneal details, targeted treatment, and treatment-related details such as total treatment duration, number of treatment breaks, and total breaks duration during photo-ablation. The eye-tracker pupil position plot was exported as an image and linked to the patient’s ID and his/her HADS-anxiety sub-score. To ensure the patient’s anonymity, the IDs were replaced by a serial number afterward.
2. According to the HADS-anxiety sub-score a class category (group) was added to the data frame assigning each patient a HADS-anxiety sub-scale (sub-score ≤ 7: normal, a sub-score of 8–10: borderline, and a sub-score of 11–21: case).
3. The patients’ age at the time of operation and the planned treatment spherical equivalent of the studied eye were automatically calculated and organized in the data frame. Also, we calculated the treatment (active photo-ablation) duration in cases with treatment breaks by subtracting the break duration from the total duration.
4. Every eye-tracker PC position plot summarizes the captured PC locations relative to the center of the tracking area (centration point) in the horizontal (x) and vertical (y) directions. The tracking area is a square with dimensions of 6 × 6 mm (3 mm above and below the center and 3 mm temporal and nasal). Each sampled PC position is marked at the corresponding coordinate by a small blue square that may overlap. The plot does not include transparencies to enhance the visualization of overlapping squares. Samples of these plots are shown in Fig. 1.

**Fig. 1:**
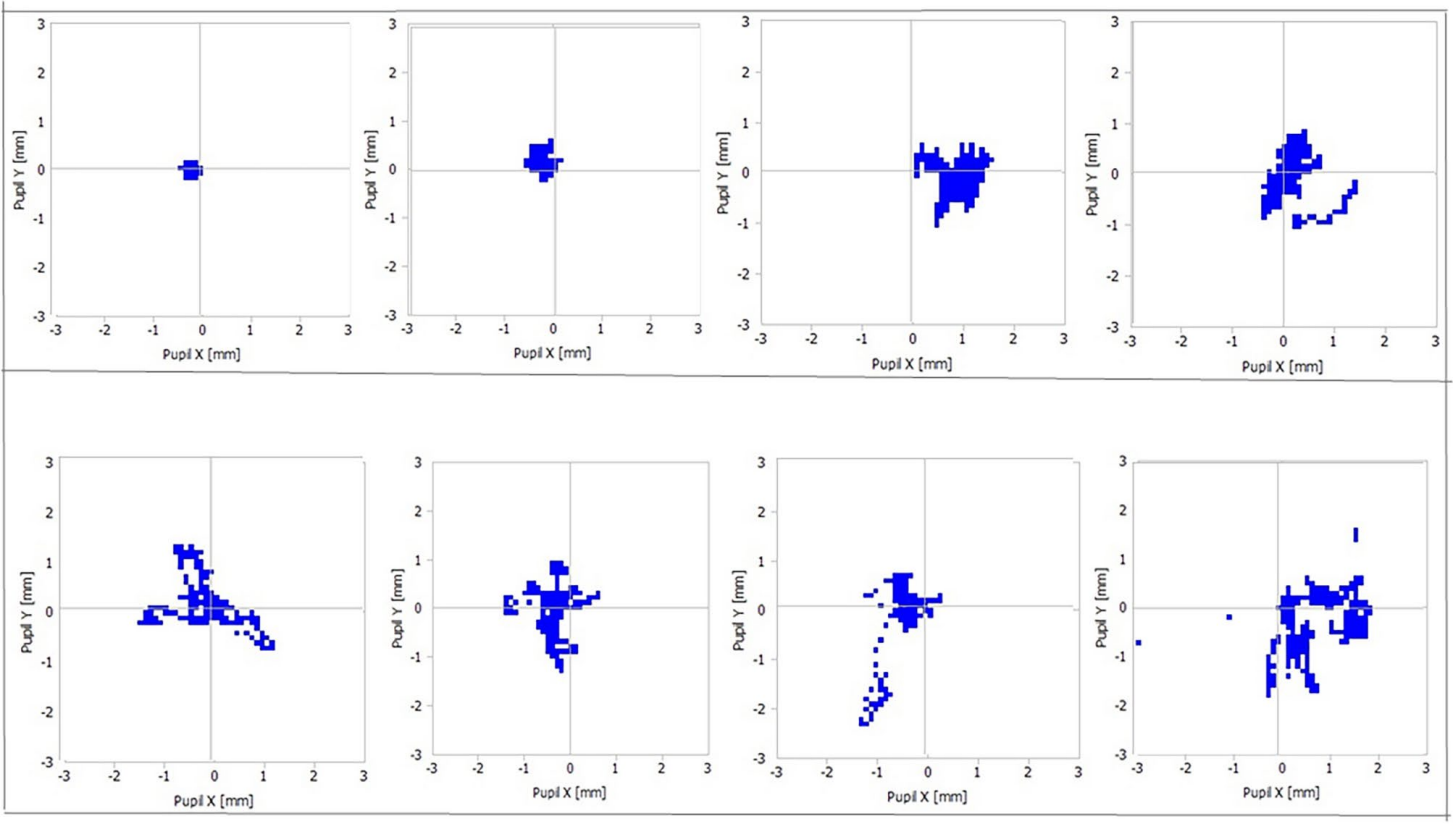
Eye tracker’s pupil center plot. Samples of eye tracker’s pupil center plots exported from the WaveLight EX500.
5. We extracted three parameters to numerically describe the variable shape of the tracked PC position marks:
  (A) Sum area (SA): This parameter represents the total area of all the blue squares in the plot.
  (B) Sum perimeter (SP): this parameter represents the sum of perimeters of all blue squares clusters in the plot.
  (C) Average centroid deviation (ACD): This parameter is calculated by first finding the center of each blue square cluster. Then, the distance between each center and the center of the tracking area is calculated. This distance is then weighted by the area of the square cluster, with larger clusters having a greater weight. Finally, the weighted distances are averaged to get the ACD. Figure 2 shows some examples of these plots after the centroids have been defined.

**Fig. 2:**
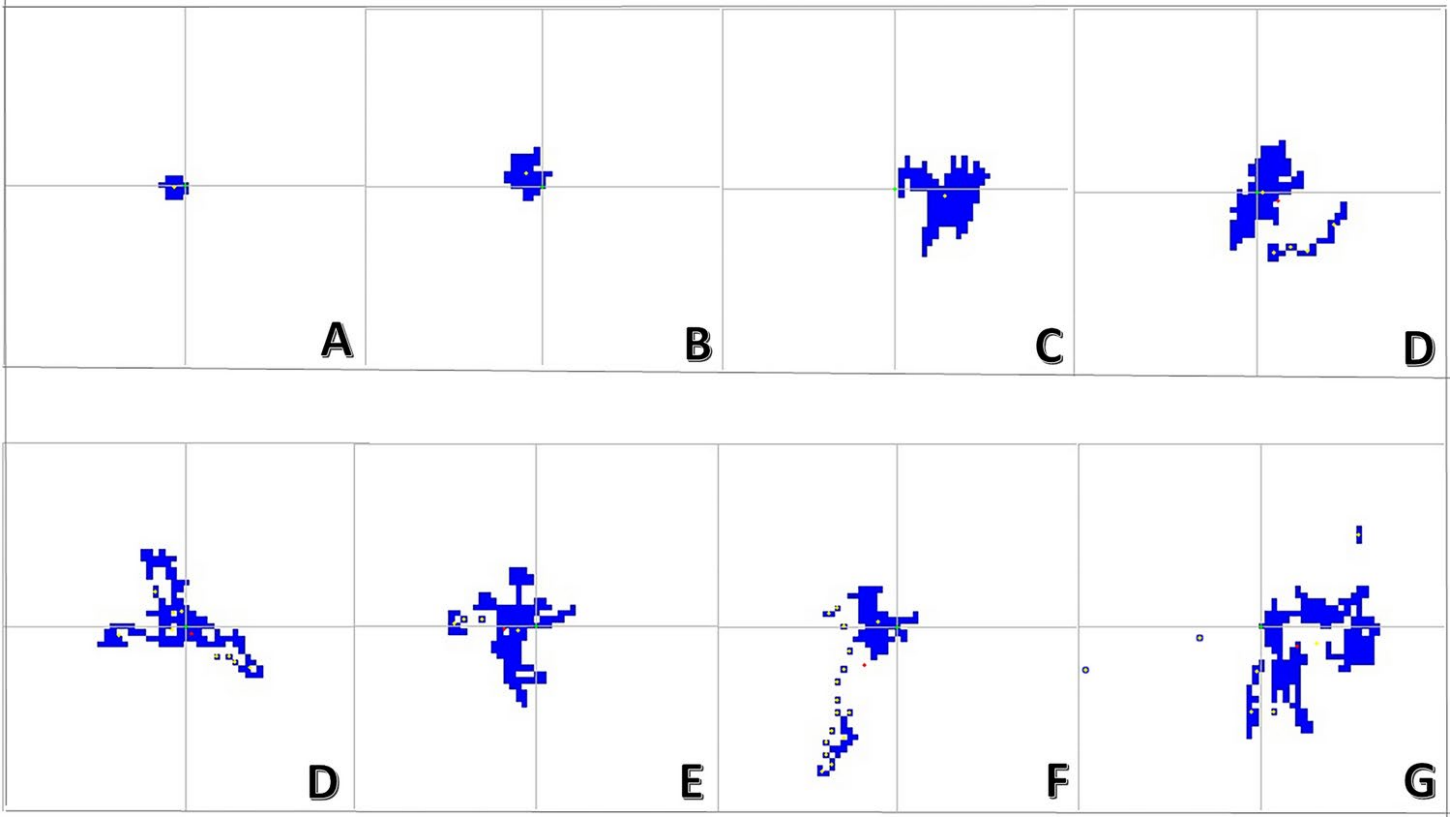
Centroids in eye-tracker’s pupil center plots. The same plot samples as in Fig. 1, after defining centroids using a weighted average algorithm. Centroids of isolated islands formed by confluent pupillary center position marks are marked with yellow rhombi, and the calculated weighted average centroid position of multiple islands is marked with a red rhombus.
6. We used linear regression to combine the three parameters (SA, SP, and ACD) into a single score that was related to anxiety, as measured by the HADS-anxiety subscale. The following formula was produced by this approach:
  $${\text{Summary}}\,{\text{score}} = 0.005*{\text{SA}} + 0.027*{\text{SP}} + 0.213*{\text{ACD}} + 4.458$$

This summary score will be referred to as the fixation instability score (FIS) afterward.

### Correlations

**Fig. 3:**
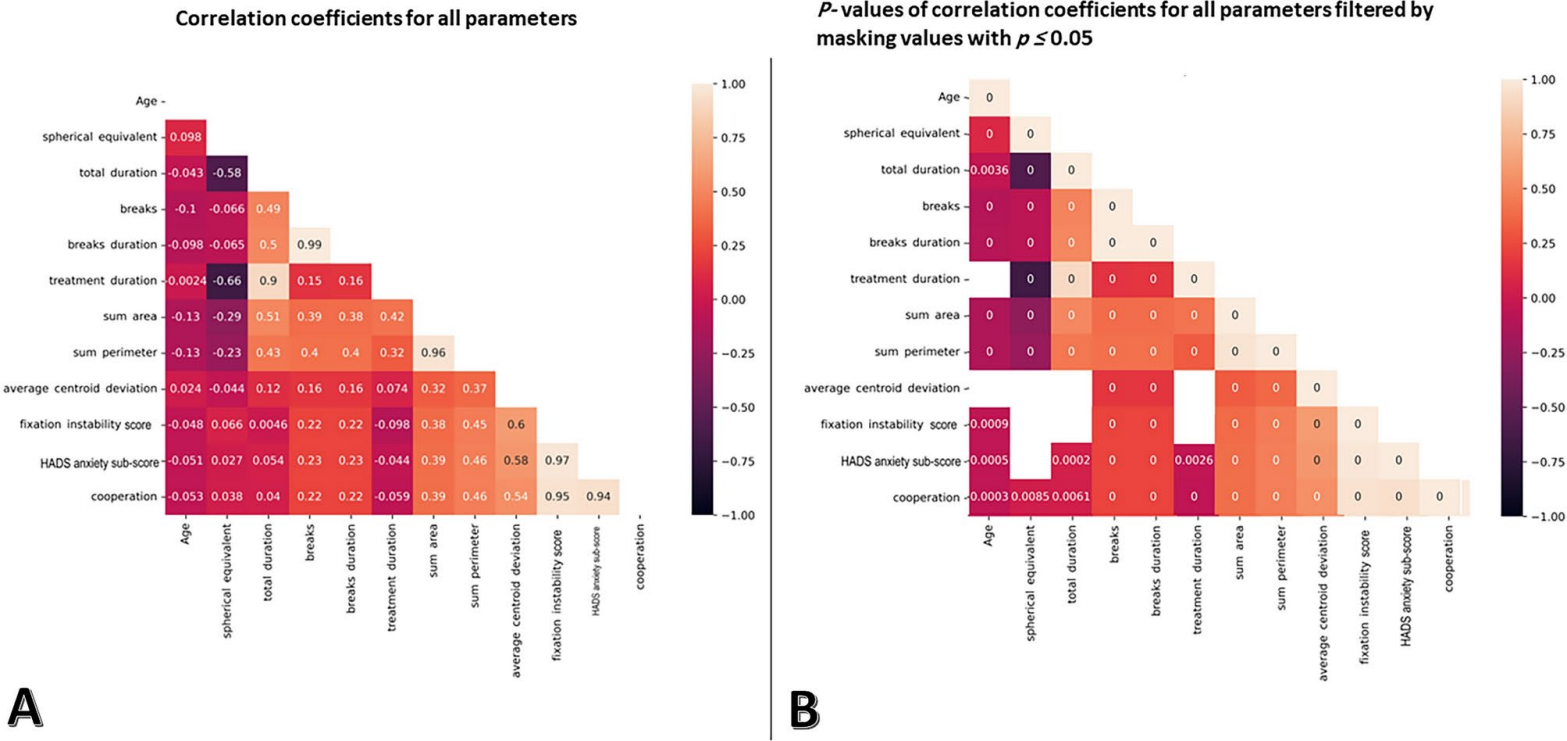
Spearman rank correlation of the studied parameters. Heatmaps displaying Spearman rank correlation coefficients of all parameters before (**A**) and after (**B**) filtering for statistically significant correlations with *p* value ≤ 0.05. HADS = Hospital Anxiety and Depression Scale.

A pairwise correlation using the Spearman rank correlation coefficient was used to investigate the association between these eye-tracker plot-generated parameters, FIS, the HADS-anxiety sub-score and other characteristics as illustrated in Fig. 3.

### Classification performance

To compare the performance of the eye-tracker plot-generated parameters (SA, SP, and ACD), and the FIS in classifying patients into three groups based on their HADS-anxiety subscale scores, we used four support vector machine^9^ (SVM) classifiers. Each SVM was trained on a randomly sampled 70% of the dataset using one of the parameters (SA, SP, ACD, or FIS) and then tested on the remaining 30% of the dataset. To evaluate the performance of each SVM, we used a one-vs-one multiclass approach to plot the receiver operating characteristic (ROC) curve and calculate the mean area under the curve (AUC)^10^. We also used each parameter to train a binary SVM classifier for each class pair. This allowed us to compare the ROC curves of SA, SP, ACD, and FIS using the method described by DeLong et al.^11^. Finally, we used Youden’s J statistic to calculate the optimal cutoff point for the FIS in distinguishing between the three groups.

### Average class-wise PC positions map

To provide an insight into the most frequently spotted PC off-center positions captured by the eye tracker during photoablation, we employed principal component analysis (PCA) for dimensionality reduction to generate an average class-wise PC positions map of the right and left eye using all available maps of each class as categorized by the HADS anxiety sub-scale.

### Statistical analysis

We used the Python programming language (version 3.9.13) to write scripts for our statistical analyses^12^. We performed all our statistical analyses using the Scipy (Scientific Computing tools for Python, version 1.8.1) and scikit-learn (version 1.1.1) libraries for Python. Scikit-learn is a Python module for machine learning that is built on top of Scipy^13^.

Descriptive statistics were used to analyze the baseline characteristics of patients in the three HADS-anxiety sub-scale classes. Normally distributed data were expressed as the mean plus or minus the standard deviation. We used the Kolmogorov–Smirnov 1-sample test to check the probability distribution of each parameter for goodness of fit. We used the chi-square test to compare the gender distribution of the groups. One-way ANOVA test was used to analyze parameters with normal distribution, and the Kruskal–Wallis test to compare the medians of the three HADS-anxiety subscale groups. Pairwise Wilcoxon rank-sum tests was utilized to determine which groups were different from each other. We considered a p-value of 0.05 or less to be statistically significant.

We used NumPy, a Python library for scientific computing, to calculate the sample size needed for our study. Given a sample size of 2367 (789 for each group), based on a two-sided alpha of 0.05 and a power of 0.90, our minimum detectable change in anxiety was 0.2. Selecting a high statistical power was necessary to provide robustness to the newly developed scale.

## Results

A total of 2582 eligible participants were included in the study. Of them, 57 patients canceled the surgery, 38 declined to participate on the day of surgery, 29 asked for sedation before surgery, 12 were unable or had too much difficulty answering the HADS-Anxiety questionnaires, and 11 were retroactively excluded due to the occurrence of flap complication or gas bubbles in the anterior chamber, failure of eye-tracking during ablation, or the surgeon asked to manually inactivate the eye tracker. Thus, a total of 2435 participant’s eyes were available for analysis. The HADS-Anxiety questionnaire was administered to participants 29.5 ± 8.2 min before surgery. The average participant age was 33.32 ± 8.41 years, and 55.2% of the participants were female. Stratification of sex for the HADS anxiety sub-scale revealed a statistically significant difference in the number of males and females in the case group (*p* < 0.001), but not in the normal or borderline groups (*p* = 0.477 and *p* = 0.056, respectively). Stratification of age, spherical equivalent, total duration, and treatment duration for the HADS anxiety sub-scale revealed no statistically significant difference between sub-scale groups. The mean HADS-anxiety sub-score was 5.5 ± 5.2. Stratification by the HADS-anxiety scale revealed that the mean HADS-anxiety scores were 2.7 ± 1.2, 9.1 ± 0.4, and 15.2 ± 2.6 for the normal, borderline, and case groups, respectively. The mean cooperation VAS scores for the normal, borderline, and case groups were 2.3 ± 0.8, 4.1 ± 0.4, and 5.3 ± 0.3, respectively.

Heart rate measurements differed significantly between the three HADS anxiety sub-scale groups at all time-points (*p* < 0.001). There was a clinically significant increase in heart rate (HR) from baseline to ablation time in the case group (*p* = 0.011). There was also a clinically significant increase in HR from flap creation time to ablation time in the borderline and case groups (*p* = 0.020, *p* = 0.029, respectively). Additionally, there was a clinically significant decrease in heart rate from ablation time to the end of surgery time point in the borderline group (*p* = 0.009). Pairwise comparisons of the medians of break durations in the three anxiety subscale groups were statistically significant (*p* < 0.001), except for the comparison between the normal and borderline groups (*p* = 1.00). Analysis of the number of breaks during ablation showed no statistically significant differences. The mean time needed for calculating the SA, SP, ACD, and FIS from individual PC tracking plots was 5.17, 5.19, and 5.23 ms for the normal, borderline, and case groups respectively. Table 1 summarizes the demographic characteristics and baseline studied parameters of the study participants.

**Table 1:** Baseline characteristics by HADS anxiety-subscale (classes).

Parameter	Normal (n = 1007)	Borderline(n = 562)	Case (n = 866)	*p* value
Eye (right/left)	534/473	274/288	443/423	0.263
Gender (male/female)	501/506	289/273	388/478	**< 0.001**
Age (years)				
Median	33.00	33.00	32.00	0.488
IQR	11	12	11.5	
Range	18, 66	18, 62	18, 66	
Spherical equivalent				
Median	− 3.75	− 3.62	− 3.25	0.176
IQR	3.70	3.76	3.83	
Range	− 14.00, 7.25	− 13.00, 7.12	− 13.00, 7.62	
Total duration (s)				
Median	11.00	10.0	10.0	0.568
IQR	7.00	8.00	8.00	
Range	1.00, 351.00	1.00, 132.00	1.00, 231.00	
Number of breaks				
Median	0.00	0.00	0.00	0.224
IQR	0.00	0.00	1.00	
Range	0.00, 6.00	0.00,7.00	0.00, 8.00	
Breaks duration (s)				
Median	0.00	0.00	0.00	**0.020**
IQR	0.00	0.00	4.00	
Range	0.00, 25.00	0.00, 11.00	0.00, 21.00	
Treatment duration (s)				
Median	11.00	10.00	10.00	0.568
IQR	7.00	7.00	7.00	
Range	1.00, 24.00	1.00, 23.00	2.00, 22.00	
Mean HR ± SD, (range)				
Baseline	65.5 ± 2.2, (61,71)	65.4 ± 1.6, (61,76)	69.8 ± 3.2, (62,79)	**0.009**
At the start of the operation	67.0 ± 2.3, (63,75)	67.5 ± 3.1, (62,79)	74.9 ± 5.0, (67,88)	**0.001**
During flap creation	66.1 ± 2.1, (62,71)	67.3 ± 1.4, (62,74)	75.5 ± 3.6, (65,86)	**< 0.001**
During ablation	68.5 ± 2.0, (64,82)	71.5 ± 3.5, (66,81)	79.5 ± 6.2, (64,98)	**0.007**
At the end of surgery	68.0 ± 1.8, (64,83)	70.0 ± 2.2, (66,82)	79.9 ± 4.6, (65,92)	**< 0.001**
SA				
Median	490.50	601.75	1007.00	**< 0.001**
IQR	316.625	430.0	650.5	
Range	46.00, 2759.00	97.00, 3339.00	152.00, 5297.00	
SP				
Median	110.00	134.38	225.01	** < 0.001**
IQR	43.07	65.31	121.31	
Range	35.31, 471.74	43.07, 772.65	53.07, 1037.76	
ACD				
Median	12.08	20.03	26.57	**0.002**
IQR	9.75	13.29	14.72	
Range	0.00, 78.92	1.0, 77.59	1.0, 98.01	
FIS				
Median	11.00	32.00	41.00	**< 0.001**
IQR	3.00	4.00	5.00	
Range	0.00, 82.00	9.00, 55.00	33.00, 90.00	
Cooperation VAS score				
Median	2.00	4.00	5.00	**0.02**
IQR	1.00	0.00	1.00	
Range	1.00, 8.00	2.00, 6.00	4.00, 10.00	
HADS anxiety sub-score				
Median	3.00	9.00	16.00	**< 0.001**
IQR	2.00	1.00	3.00	
Range	0.00, 7.00	8.00, 10.00	11.00, 21.00	

ACD, average centroid deviation; FIS, fixation instability score; HADS, Hospital Anxiety and Depression Score; HR, heart rate (beats/ min); IQR, Interquartile range after removing outliers; n, number of subjects; SA, sum area; SP, sum perimeter; VAS, visual analog scale.

Normal = participants with HADS anxiety sub-score between 0 and 7; Borderline = participants with HADS anxiety sub-score between 8 and 10; Case = participants with HADS anxiety sub-score between 11 and 21.

Bold type signifies *p* ≤ 0.05.

In Spearman rank correlation analysis, the FIS was strongly correlated with the HADS anxiety sub-score (r = 0.97, *p* < 0.001) and the cooperation VAS score (r = 0.95, *p* < 0.001). Strong correlations were also found between SA and SP (r = 0.96, *p* < 0.001) and between the number of breaks and break duration (r = 0.99, *p* < 0.001) (Fig. 3).

**Fig. 4:**
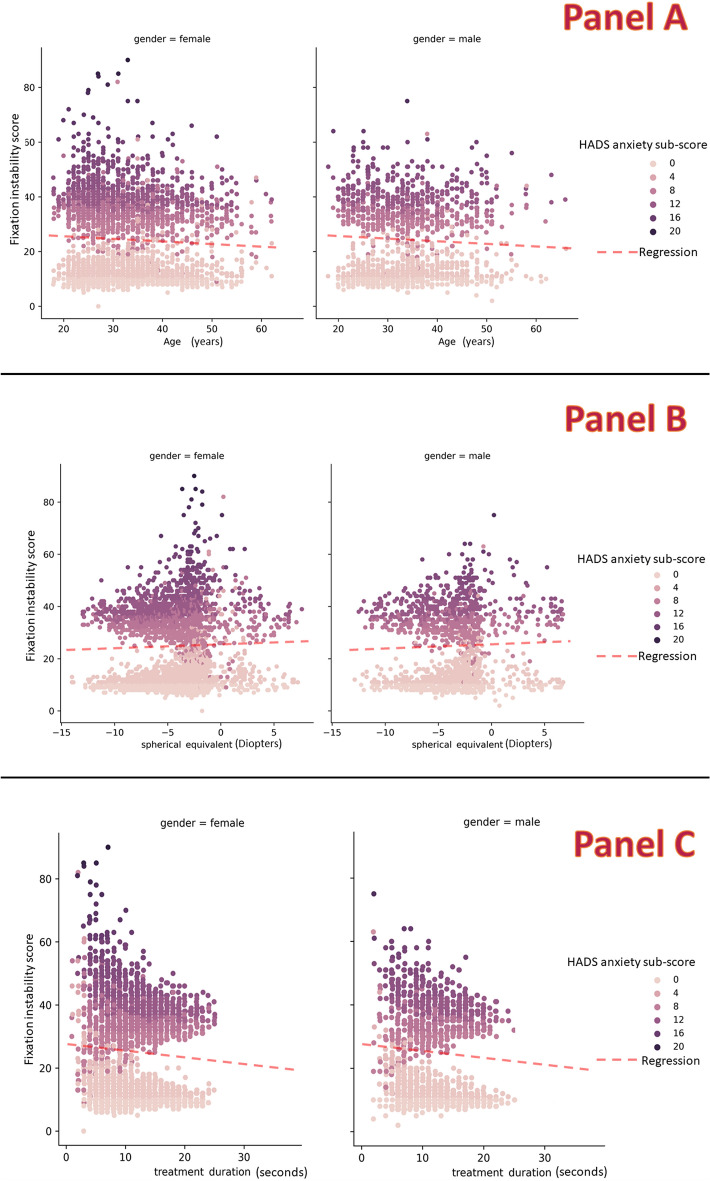
Relationship between fixation instability score, HADS anxiety sub-score with age, spherical equivalent and treatment duration. Combined categorical (male/female) and facet grid plots showing the relationship between fixation instability score, Hospital Anxiety and Depression Scale (HADS) anxiety sub-score with age (**A**), spherical equivalent (**B**), and treatment duration (**C**), respectively. The dashed lines indicate the observed regression.

Figure 4 shows how the distribution of FIS varies with age, spherical equivalent, and treatment duration, stratified by gender.

**Fig. 5:**
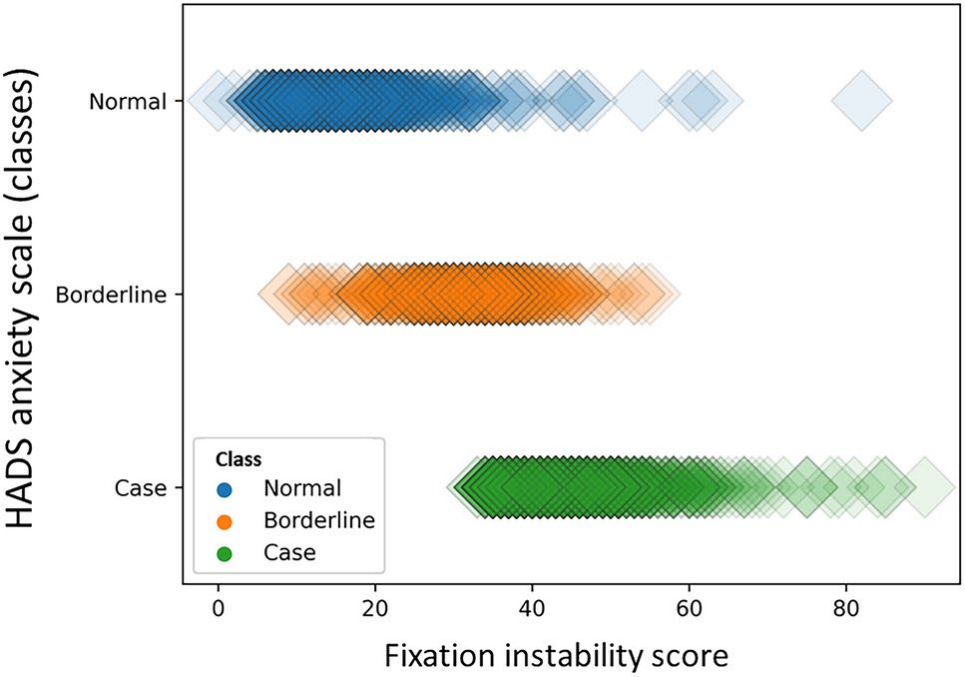
Fixation instability scores among the 3 groups of HADS anxiety sub-score Legend: The distribution of fixation instability scores between normal, borderline, and case groups (classes) of the Hospital Anxiety and Depression Scale anxiety subscale.

Figure 5 shows the distribution of FIS between the three HADS anxiety groups.

**Fig. 6:**
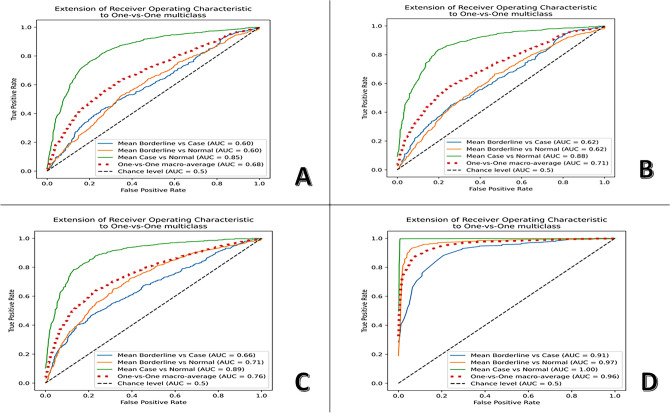
Receiver operating characteristic (ROC) diagrams. Extension of receiver operating characteristic (ROC) to one-vs-one multiclass for 3 Hospital Anxiety and Depression Scale anxiety subscale groups (normal, borderline, and case) in the test subset. (**A**) ROC results for support vector machine (SVM) using the Sum Area parameter, (**B**) ROC results for SVM using the Sum perimeter parameter, (**C**): ROC results for SVM using the Average Centroid deviation parameter, (**D**) ROC results for SVM using the Fixation instability score.

Figure 6 shows ROC curves for four SVM models, each trained on a different PC tracking plot parameter (SA, SP, ACD, and FIS) to classify the three HADS anxiety groups in a 30% test set. The AUC for SA, SP, ACD, and FIS using a one-vs-one multiclass approach is shown in Table 2. Pairwise AUC comparisons for SA, SP, ACD, and FIS using DeLong’s method are shown in Table 3.

**Table 2:** Receiver operating characteristic analysis with the area under the curve.

Parameter	One-versus-one	Positive class	AUC	Mean AUC ± SE
Sum Area	Borderline versus case	Borderline	0.41	0.57 ± 0.016
		Case	0.73	
	Borderline versus Normal	Borderline	0.63	0.56 ± 0.015
		Normal	0.59	
	Case versus Normal	Case	0.82	0.82 ± 0.002
		Normal	0.82	
Sum Perimeter	Borderline versus case	Borderline	0.31	0.50 ± 0.016
		Case	0.69	
	Borderline versus Normal	Borderline	0.59	0.59 ± 0.015
		Normal	0.59	
	Case versus Normal	Case	0.77	0.77 ± 0.011
		Normal	0.77	
Average centroid Deviation	Borderline versus case	Borderline	0.41	0.50 ± 0.016
		Case	0.59	
	Borderline versus Normal	Borderline	0.67	0.67 ± 0.013
		Normal	0.67	
	Case versus Normal	Case	0.74	0.74 ± 0.012
		Normal	0.74	
Fixation Instability Score	Borderline versus case	Borderline	0.92	0.93 ± 0.008
		Case	0.94	
	Borderline versus Normal	Borderline	0.97	0.98 ± 0.004
		Normal	0.99	
	Case versus Normal	Case	1.00	1.00 ± 0.000
		Normal	1.00	

AUC: area under the curve; SE: standard error.

Normal = participants with HADS anxiety sub-score between 0 and 7; Borderline = participants with HADS anxiety sub-score between 8 and 10; Case = participants with HADS anxiety sub-score between 11 and 21.

The standard error of the AUC score was computed using its equivalence to the Wilcoxon statistic.

**Table 3:** Pairwise comparisons of the area under the curve for SA, SP, ACD, and FIS.

Classes/parameters	Mean ∆ AUC ± SE	95% CI	Z statistic	*p* value
Normal versus Borderline				
SA & SP	0.027 ± 0.001	0.017, − 0.072	− 2.14	**0.016**
SP & ACD	0.094 ± 0.001	− 0.054, − 0.134	− 2.49	**0.006**
SA & ACD	0.121 ± 0.002	− 0.077, − 0.166	− 3.97	**< 0.001**
SP & FIS	0.357 ± 0.010	− 0.319, − 0.394	− 21.33	**< 0.001**
ACD & FIS	0.263 ± 0.011	− 0.228, − 0.298	− 9.53	**< 0.001**
SA & FIS	0.384 ± 0.009	− 0.344, − 0.424	− 11.57	**< 0.001**
Borderline versus Case				
SA & SP	0.040 ± 0.001	− 0.001, − 0.079	− 2.17	**0.015**
SP & ACD	0.001 ± 0.001	0.037, − 0.038	0.40	0.657
SA & ACD	0.038 ± 0.001	− 0.002, − 0.079	− 2.48	**0.007**
SP & FIS	0.159 ± 0.012	− 0.138, − 0.180	− 4.39	**< 0.001**
ACD & FIS	0.158 ± 0.012	− 0.138, − 0.179	− 4.21	**< 0.001**
SA & FIS	0.199 ± 0.013	− 0.177, − 0.222	− 5.97	**< 0.001**
Normal versus Case				
SA & SP	0.030 ± 0.001	− 0.006, − 0.054	− 1.97	**0.025**
SP & ACD	0.022 ± 0.001	− 0.001, − 0.043	− 0.02	**0.044**
SA & ACD	0.052 ± 0.002	− 0.029, − 0.075	− 2.52	**0.006**
SP & FIS	0.120 ± 0.007	− 0.103, − 0.137	− 9.84	**< 0.001**
ACD & FIS	0.098 ± 0.006	− 0.082, − 0.113	− 9.52	**< 0.001**
SA & FIS	0.150 ± 0.008	− 0.130, − 0.169	− 11.57	**< 0.001**

∆ AUC, difference between the area under the curve; ACD, average centroid deviation; CI, confidence interval; FIS, fixation instability score; SA, sum area; SE, standard error; SP, sum perimeter.

Normal = participants with HADS anxiety sub-score between 0 and 7; Borderline = participants with HADS anxiety sub-score between 8 and 10; Case = participants with HADS anxiety sub-score between 11 and 21. Bold type signifies *p* ≤ 0.05.

**Fig. 7:**
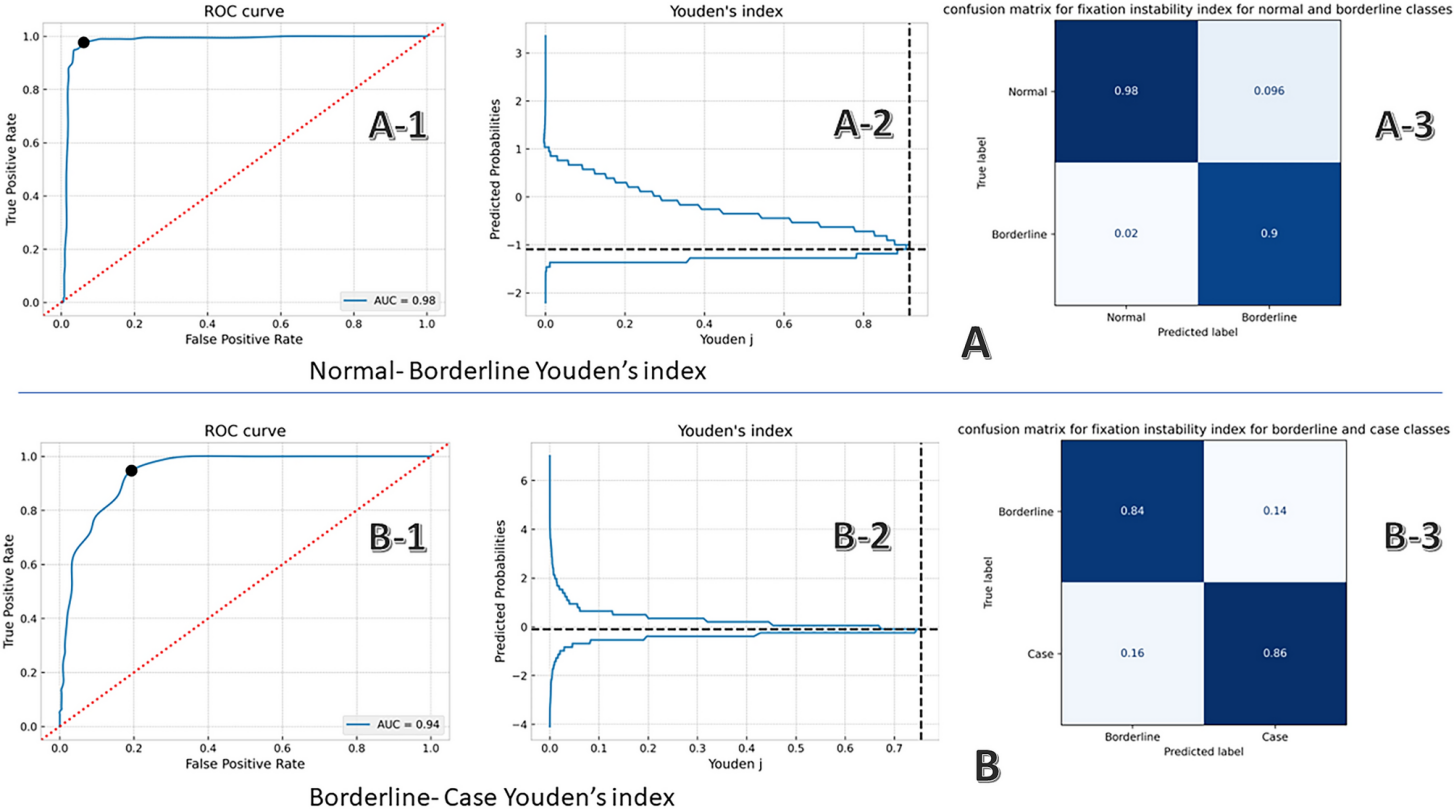
Support vector machine (SVM) classifiers. (**A**) Results for binary (normal vs. borderline) classification task by a trained support vector machine (SVM) on the test subset using the Fixation Instability score (FIS). A-1: Receiver operating characteristic (ROC) curve with an area under the curve (AUC) of 0.98. The ROC curve is marked by a black dot at the site closest to the perfect classification point. A-2: Plot showing probability score at the cut-off point of 12 and the Youden Index. A-3: The corresponding confusion matrix. (**B**) Results for binary (borderline vs. case) classification task by a trained SVM on the test subset using the FIS. B-1: ROC curve with an AUC of 0.94. The ROC curve is marked by a black dot at the site closest to the perfect classification point. B-2: Plot showing probability score at the cut-off point of 36 and the Youden Index. B-3: The corresponding confusion matrix.

The SVM model using FIS was the best-performing classifier having an accuracy of 92% on the test set and a one-vs-one macro-average AUC of 0.9. For FIS the precision, sensitivity, specificity, and F1 scores for the normal class were 96%, 97%, 96%, and 97%, respectively; for the borderline class, 81%, 74%, 94%, and 77%, respectively; and for the case class, 85%, 89%, 92%, and 87%, respectively. Two binary SVM classifiers were implemented for defining the cut-off values of the FIS for the HADS anxiety sub-scale groups. For the first SVM classifier (normal-vs- borderline), the Youden index was highest with 0.92 at a threshold of 12, resulting in the best separation between normal (≤ 12) and borderline groups (> 12). For the second SVM classifier (borderline-vs- case), The Youden index was highest with 0.75 at a threshold of 36, resulting in the best separation between borderline (≤ 36) and case class (> 36) eyes (Fig. 7). This resulted in the development of the fixation instability scale (0–90), with values from 0 to 12 considered normal, from 13 to 36 considered borderline, and from 37 to 90 considered anxiety cases.

**Fig. 8:**
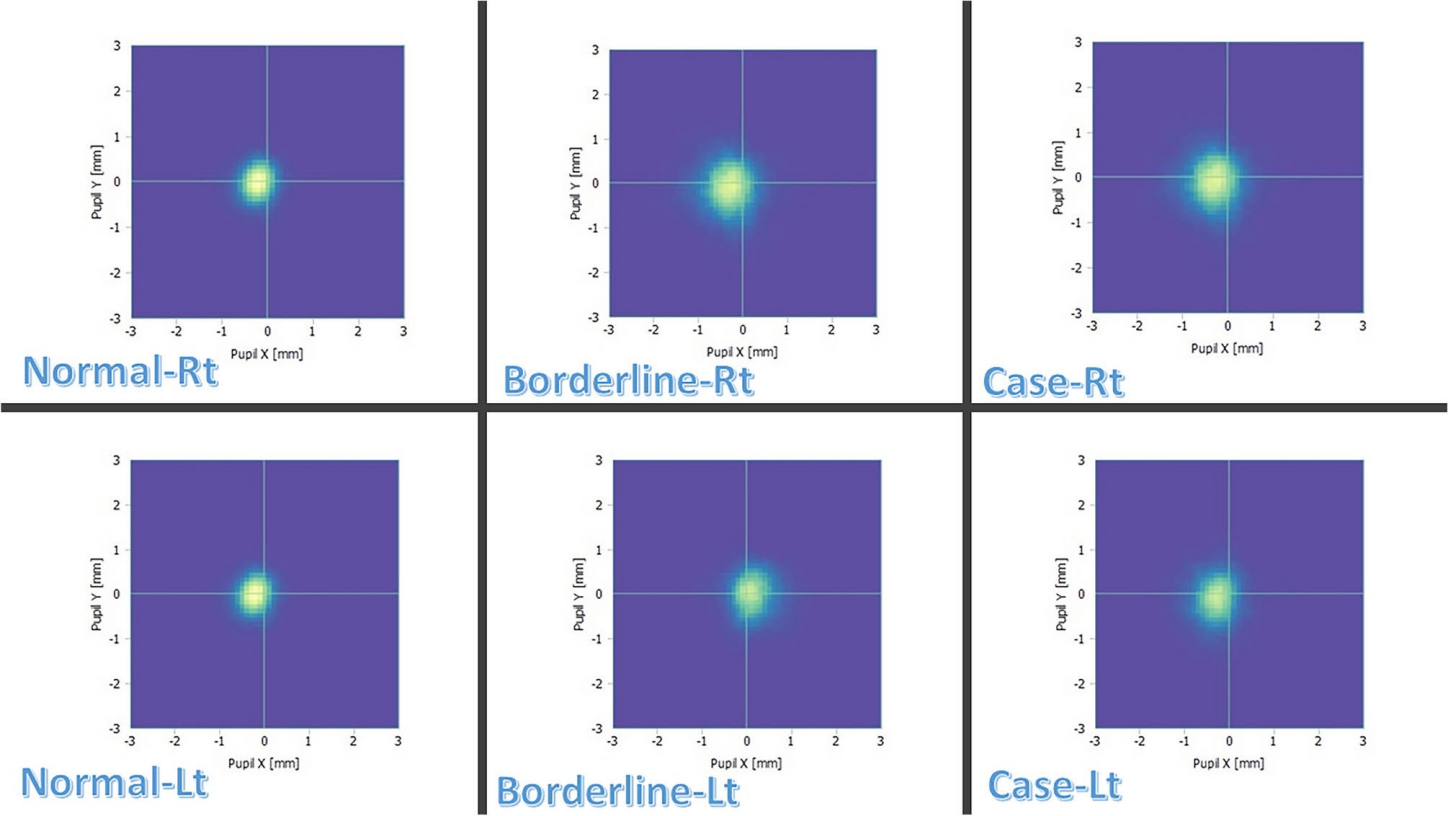
Average maps of pupil center tracking plots. Average maps of pupil center tracking plots for all right and left eyes in each Hospital Anxiety and Depression Scale anxiety subscale group (surgeon’s view). The center of the crosshair corresponds to the central fixation target. Lighter areas indicate the more commonly spotted locations of the pupil center during tracking.

Averaged PC tracking plots showed that, during LASIK ablation, the right eye PC decentered nasally in the horizontal (X) axis and upwards in the vertical (Y) axis in all groups. In the left eye, the PC decentered temporally in the X-axis in the normal and case groups and nasally in the borderline group, and upwards in the Y-axis in all groups. The area of decentration increased progressively from the normal to borderline to case HADS anxiety sub-scale groups (Fig. 8).

## Discussion

In this study of surgical anxiety among LASIK patients, we found the patients with the highest anxiety about the surgical procedure were more prone to fixation instability during photoablation were often considered uncooperative during the procedure in surgeons’ view, and had more tachycardia.

Many authors consider self-assessment questionnaires to be a sensitive and accurate way to measure anxiety^14,15^. We selected the HADS because it is a reliable instrument for evaluating depression and anxiety in hospital outpatients. It also has separate scores for depression and anxiety, is not affected by physical illness, is quick and easy to administer, and is standardized^8,16^. These qualities make it practical for use in a high-turnover laser vision correction unit. In our study, 35.5% of participants had anxiety, 23.1% had borderline anxiety, and 41.4% had no evidence of anxiety prior to LASIK surgery.

Our study observed a statistically significant difference in the sex/gender distribution within the case group, with a higher proportion of females compared to males. This finding is noteworthy, considering the overall balanced male-to-female ratio among participants. While definitive conclusions about the underlying reasons for this disparity are beyond the scope of our current investigation, it highlights the potential role of sex/gender in influencing anxiety experiences related to LASIK procedures.

Analyzing the association between age and the covariates HADS anxiety sub-score, FIS, and cooperation VAS score in our study, revealed a statistically significant negative linear association. A linear association was observed, as patient cooperation was expected to increase with age while anxiety and FIS were expected to decrease, in agreement with previous reports^17,18^.

The VAS was originally designed for self-assessment of pain^19^, but it was adapted in this study to serve as a measure of patient cooperation. This approach has been used previously for patient cooperation during phacoemulsification^20–22^. In our work, there was a large and highly significant correlation between the FIS and both HADS anxiety sub-scores and patient cooperation, emphasizing the close relationship between patient anxiety and their ability to cooperate during surgery. Although anxiety and fixation instability are highly associated, reducing anxiety with sedatives and anxiolytics may not improve fixation stability, as sedatives can affect consciousness, clarity, and reaction time^23^. This complex relationship between anxiety and fixation stability is further nuanced by research on refractive surgery in patients with psychiatric disorders. The study by Ortega-Usobiaga et al.^24^ offers a valuable perspective. Their work showed excellent surgical outcomes and high satisfaction in this population, highlighting the importance of individualized patient assessment. Our study specifically excluded patients with clinically relevant psychiatric diagnoses, as our aim was to investigate anxiety as a more common, transient experience during LASIK, and its impact on fixation stability. Furthermore, our focus was on measuring fixation instability as a potential objective marker of anxiety; thus, we have clarified our statement regarding sedatives and anxiolytics to emphasize their potential to confound intraoperative anxiety and fixation stability measurement, rather than precluding their use in specific patient populations.

Heart rate measurements increased significantly for all HADS anxiety subscales during the procedure, despite the expected decrease in HR due to the oculocardiac reflex during the suction phase for flap creation. This is likely due to pre-surgical stress, which can increase basal HR and prevent the oculocardiac reflex^25^. The difference in HR between the three groups at all time-points confirms the concordance between the subjective HADS anxiety subscales and the HR as an objective measure of anxiety level.

Although most patients undergoing LASIK maintain light perception throughout the procedure, some experience temporary vision loss during the brief flap creation stage^26,27^. Additionally, many patients report various visual sensations during surgery, with nearly 20% finding these experiences frightening^27^. Recognizing the potential anxiety these phenomena can cause, our study aimed to proactively address these concerns through exhaustive patient education. Specifically, we informed patients about the expected visual changes, sounds, and smells they might encounter during the procedure, emphasizing the temporary nature of these sensations to alleviate potential anxiety. We also stressed the importance of maintaining focus throughout the procedure to ensure optimal outcomes. To ensure unbiased results, we used the first ablated eye, so the patients did not have the advantage of prior experience that could alleviate anxiety due to uncertainty during the operation.

Previous reports have mentioned difficulties faced by patients with high myopia in visualizing the fixation light of the excimer laser^28^. However, in our study, we did not observe a significant association between high myopia and FIS. Additionally, FIS was not found to be sensitive to long ablation time in cases of high errors. This can be attributed to the method of calculating the component parameter ACD where deviations from centration in opposite meridians can offset each other, allowing the more dominant direction of decentration to be a better predictor of this parameter.

Breaks during ablation were intended to prevent corneal overheating, patient discomfort, or difficulty visualizing the fixation light, or to deactivate the laser by the surgeon if the eye moved vigorously or by the tracker if the PC moved outside the active tracking area. There was a significant correlation between longer treatments and more breaks and breaks of longer duration. However, these parameters were not used to quantify patient fixation instability, as they were confounded by surgeon preference in long treatments^4,5^.

Average maps showed that most eyes had a leftward shift, except for left eyes in the borderline group, which had a rightward shift. Additionally, most eyes had an upward shift. While these patterns are interesting, it’s important to acknowledge that variations in angle kappa between right and left eyes, as well as natural variations in X and Y axis positions, could contribute to these findings. Numerous studies have demonstrated such variations^29–31^. Additionally, the upward shift can be partially explained by Bell’s phenomenon, which occurs when a patient actively squeezes their eyelids. The predominance of the nasal/temporal shift (after accounting for the potential influence of angle kappa) cannot be fully explained by the horizontal component of Bell’s phenomenon, which is commonly temporal^32^. A possible contributing factor is the occurrence of the oculocephalic reflex, an application of the vestibulo-ocular reflex (VOR)^33^. The VOR stabilizes the retinal image during head movement by producing compensatory eye movements in the opposite direction. Anxious patients may experience slight head movements during ablation time, especially at the anxiety-provoking moment of the sudden, noisy start of each treatment. These head movements are a protective reflex. Given that a left-sided head shift is commonly followed by a right-sided corrective eye movement after a lag of 10 ms^33^, and the EX-500 eye tracker has a latency time of 2 ms (Alcon communication), the tracker can record a left-sided offset during this lag difference.

Our study has its own limitations. One limitation of our study is the lack of complete pupil center movement data throughout the entire ablation procedure. The available eye-tracking system provided data points at specific intervals during photoablation, but it did not capture a continuous tracking profile. Another potential limitation of this study is that our study involved data collection from procedures performed by multiple surgeons. Additionally, While PC plot analysis provided insights into horizontal and vertical eye movements during LASIK (used to define fixation instability), it does not capture rotational movements (cyclotorsion), which can occur during surgery due to loss of fixation, excitement, or small head rotations^34^. Furthermore, our study included both SA and SP parameters derived from the eye-tracker plot, even though they exhibited a high correlation in our analysis. While the initial correlation is noteworthy, it’s important to consider the potential added value of both parameters in future research, particularly when exploring the applicability of the FIS to more complex shapes. Finally, we utilized a validated Arabic version of the HADS provided by the copyright holders and its applicability in the context of pre-surgical anxiety in LASIK patients might not be fully optimized^35,36^.

## Conclusion

The fixation instability score and scale as useful instruments to quantify anxiety-related fixation instability during LASIK. Future studies are required to validate various perioperative stress reduction measures for LASIK and to explore the visual and refractive outcomes of LASIK in patients with various fixation instability scores. Also, this approach can be extended to other topical ocular surgeries, provided that a simple eye tracker facility is provided during the recording of the procedure.

## Data Availability

The data that support the findings of this study are not openly available due to reasons of sensitivity and are available from the corresponding author upon reasonable request.
